# Effects of a scalable home‐visiting intervention on child development in slums of urban India: evidence from a randomised controlled trial

**DOI:** 10.1111/jcpp.13171

**Published:** 2019-12-03

**Authors:** Alison Andrew, Orazio Attanasio, Britta Augsburg, Monimalika Day, Sally Grantham‐McGregor, Costas Meghir, Fardina Mehrin, Smriti Pahwa, Marta Rubio‐Codina

**Affiliations:** ^1^ Institute for Fiscal Studies London UK; ^2^ Department of Economics University College London London UK; ^3^ Center for Early Childhood Education and Development Ambedkar University Delhi India; ^4^ UCL Great Ormond Street Institute of Child Health London UK; ^5^ Department of Economics Yale University New Haven CT USA; ^6^ International Centre for Diarrhoeal Disease Research Dhaka Bangladesh; ^7^ Pratham Delhi India; ^8^ Inter‐American Development Bank Washington DC USA

**Keywords:** Child development, parent–child interaction, home visiting

## Abstract

**Background:**

An estimated 63.4 million Indian children under 5 years are at risk of poor development. Home visits that use a structured curriculum to help caregivers enhance the quality of the home stimulation environment improve developmental outcomes. However, achieving effectiveness in poor urban contexts through scalable models remains challenging.

**Methods:**

Using a cluster randomised controlled trial, we evaluated a psychosocial stimulation intervention, comprising weekly home visits for 18 months, in urban slums of Cuttack, Odisha, India. The intervention is complementary to existing early childhood services in India and was run and managed through a local branch of a national NGO. The study ran from August 2013 to July 2015. We enrolled 421 children aged 10–20 months from 54 slums. Slums were randomised to intervention or control. Primary outcomes were children's cognitive, receptive language, expressive language and fine motor development assessed using the Bayley‐III. Prespecified intent‐to‐treat analysis investigated impacts and heterogeneity by gender. Trial registrations: ISRCTN89476603, AEARCTR‐0000169.

**Results:**

Endline data for 378 (89.8%) children were analysed. Attrition was balanced between groups. We found improvements of 0.349 of a standard deviation (*SD*; *p* = .005, *stepdown p* = .017) to cognition while impacts on receptive language, expressive language and fine motor development were, respectively, 0.224 *SD* (*p* = .099, *stepdown p* = .184), 0.192 *SD* (*p* = .085, *stepdown p* = .184) and 0.111 (*p* = .385, *stepdown p* = .385). A child development factor improved by 0.301 *SD* (*p* = .032). Benefits were larger for boys. The quality of the home stimulation environment also improved.

**Conclusions:**

This study shows that a potentially scalable home‐visiting intervention is effective in poor urban areas.

## Introduction

A total of 250 million children under five in low‐ and middle‐income countries (LMICs) are at risk of not reaching their developmental potential with more at‐risk children, a total of 63.4 million, in India than in any other country (Lu, Black, & Richter, 2016). Rapid urbanisation in LMICs, including India, means that almost half of the world's children now live in urban areas (UNICEF, 2013). While *on average* urban children have superior living conditions and better access to services than children in rural areas, children growing up in urban slums have educational and health outcomes that are often equivalent to, or worse than, their rural peers (Pörtner & Su, 2018; UNICEF, 2013). Lack of stimulation is a key risk factor for poor child development (Black et al., 2017) and urban slums present particular challenges: the lack of safe outdoors play spaces combined with overcrowded housing means children often have few opportunities for stimulating play (Lester & Russell, 2010).

Efficacy trials show that psychosocial interventions in LMICs that use structured curricula to encourage caregivers to engage in stimulating play and responsive interactions with young children can be successful at mitigating developmental deficits (Aboud & Yousafzai, 2015; Baker‐Henningham & Lopez Boo, 2010; Barnett, 2011; Britto et al., 2017; Engle et al., 2011; Nores & Barnett, 2010). Despite disadvantaged children and their caregivers having many and diverse needs, tightly focused interventions might be more effective at promoting child development than more comprehensive services (Barnett, 2011). Earlier trials of the tightly focused Jamaican home‐visiting model, Reach Up and Learn, found short‐term benefits to child development in Jamaica, Bangladesh and Colombia (see Grantham‐McGregor & Smith, 2016 for a recent overview). While in Colombia benefits had faded out after two years, in Jamaica effects were found well into adulthood (Andrew et al., 2018; Grantham‐McGregor & Smith, 2016).

Despite the promising evidence from efficacy trials, there is little verification of whether the benefits of such models can be maintained when implemented in a scalable manner either by governments or NGOs. Scalability depends on cost, on whether key staff can be recruited locally in sufficient numbers and on how the model fits into the existing structures of the NGO or government system who would implement it at scale. Potentially scalable delivery models have been evaluated in Colombia and Pakistan. In Colombia, home visits delivered by locally elected community leaders led to small‐to‐medium short‐term benefits but no medium‐term benefits to child development (Andrew et al., 2018; Attanasio et al., 2014). In Pakistan, both short‐ and medium‐term benefits were found from government health workers delivering a stimulation intervention although the study's design was limited by only having four units of randomisation (Yousafzai et al., 2016; Yousafzai, Rasheed, Rizvi, Armstrong, & Bhutta, 2014).

There is a lack of evidence on interventions in urban areas or from India, which has the greatest number of children at risk of poor development. Indeed, the only previous home‐visiting study from India was set in rural areas. It found positive effects on child development from delivering simple messages to mothers regarding feeding and play practices (Vazir et al., 2013).

This effectiveness trial aimed to assess the effect of home visits on child development in the urban slums of Cuttack, Odisha, one of India's poorest states. It was designed as a first step towards developing an effective and scalable early childhood intervention in India that would complement existing services.

The intervention was run and managed by the Cuttack branch of Pratham, India's largest educational NGO which works in 20 of India's 29 states. Employees from the local office were trained in the intervention, and subsequently, recruited, trained, managed and supported local women to deliver the programme. Home visitors were drawn from an abundant local labour pool; the only qualification that we listed as desirable was that visitors had completed high school, a criterion that 40% of working‐aged women in urban Odisha meet, of which just 19% currently participate in the labour force. In practice, this criterion was sometimes waived to prioritise communication skills and a positive attitude to working with families and children.

We designed the intervention to complement India's Integrated Child Development Services (ICDS) which has a network of 1.4 million Anganwadi centres providing nonformal preschool education for over‐threes. Although Anganwadi workers perform home visits to monitor young children's physical health, there is very little emphasis on psychosocial stimulation for under‐threes (Chudasama et al., 2014). Since disadvantaged children show large developmental deficits by age three, we hypothesised that there may be benefits from complementing existing services for children under three with home visits promoting psychosocial stimulation. The intervention's target age range was chosen so children could graduate into existing ICDS preschools.

Hypotheses and methods were prespecified in a pre‐analysis plan held in the trial registries. Our primary hypotheses were that the programme would improve children's cognition, language and fine motor development. Secondary hypotheses were that the programme would improve knowledge of child development and the quality of the home environment. We additionally hypothesised that, given the prevalence of son preference in Northern India (Barcellos, Carvalho, & Lleras‐Muney, 2014), impacts would differ by children's gender.

## Methods

### Study design and participants

This study was a cluster randomised controlled trial run in 54 peri‐urban slums (clusters) of Cuttack where Pratham worked. Clusters were well‐defined communities, separated by roads and waterways; the average distance between a cluster and its nearest neighbour was 0.4 km. The study's inclusion criteria were children aged 10–20 months at baseline, excluding twins and children with physical or mental disabilities. In August/September 2013, we identified eligible children through a door‐to‐door census. If fewer than seven eligible children were identified in a cluster, we added adjacent areas. When there was between seven and nine eligible children in a cluster, we included them all. When there were ten or more, we randomly selected nine for inclusion. Wherever possible, we randomly selected replacements from census lists if selected children were outside of the target age range by the time of baseline (because of delays in commencing data collection), or had relocated, or caregivers refused to participate.

Clusters were randomised by the research team in equal numbers to treatment and control using computer‐generated random numbers in October 2013. Randomisation was stratified by the number of eligible children identified through the census: fewer than nine (21%); more than nine (66%); and where adjacent areas were added (13%).

Baseline data were collected over November/December 2013, that is one to two months after randomisation, and the intervention began immediately afterwards. Endline data on child development were collected after the intervention ended in May/June 2015, 20 months after randomisation, followed by a household survey.

The process of enrolment was blind to treatment allocation, as were the testers and interviewers. Moreover, participants were not told about the intervention until after the baseline survey. While masking of participants in the treatment group was not possible, participants in the control group were unaware they were part of a control group.

The trial was registered with the ISRCTN registry (ISRCTN89476603) and the AEA registry (AEARCTR‐0000169).

### Ethical considerations

The study was reviewed and approved by the research ethics committees of University College London, UK (2168/001) and of the Institute for Financial Management and Research, India (IRB00007107). Caregivers provided written informed consent before study participation.

### Intervention

The psychosocial stimulation intervention comprised 18 months of weekly, hour‐long home visits with the target child and primary caregiver, who was usually the child's mother. The visits aimed to increase and improve maternal–child interactions and the mother's ability to promote her child's development through play. Based on the Reach Up and Learn model (Grantham‐McGregor & Walker, 2015), the intervention followed a structured curriculum of developmentally appropriate activities using low‐cost homemade toys and picture books—all adapted to the Odisha context. During visits, home visitors (HVs) demonstrated play activities and mothers practised them. Emphasis was placed on developing good relationships with mothers, encouraging them to continue the activities between visits, to respond to their children's actions and verbalisations and to celebrate their successes.

Three Pratham employees (mentors) with field experience but no specific background in child development were trained for three weeks in key child development principles, the curriculum and methods for supervising and supporting HVs. The mentors then recruited and trained 27 HVs (one per treatment slum) for four weeks, followed by two subsequent two‐to‐three days refresher trainings. The HVs were local women and most lived in the communities where they worked. They were 18–55 years old (30 on average) and 74.1% had completed high school. Mentors held weekly meetings with the nine HVs for whom they were responsible to discuss children's progress and reinforce intervention messages. They observed one visit per HV per week. A psychologist supported intervention activities throughout.

Over the 18‐month period, the intervention cost USD 251 per child allocated to the treatment group (details in Table S1). This per‐child cost would likely reduce if the intervention reached more children due to economies of scale in overheads and training.

### Outcomes and data collection

The four preregistered primary outcomes were children's cognitive, receptive language, expressive language and fine motor development, measured on the Bayley Scales of Infant and Toddler Development, third edition (Bayley‐III; Bayley, 2006). Children were tested at endline in community centres with their caregivers present. The testers had degrees in psychology or related disciplines and were trained for six weeks. At baseline, child development was measured in the home using a modified (details in Andrew et al., 2015) version of the Ages and Stages Questionnaires, third edition (ASQ‐3; Squires and Bricker, 2009).

Both the Bayley‐III and the ASQ‐3 were translated to Oriya, and words and images were adapted and piloted by Indian child development experts to aid functional equivalence and cultural relevance. Adaptations were based on those previously developed for Bangladesh. Test scales correlated significantly with each other, with children's age and socio‐economic characteristics; the Bayley‐III scales showed good internal consistency (Cronbach's α = [.84–.90]).

We internally standardised the Bayley‐III and the ASQ‐3 raw scores to remove age and tester effects and construct *Z*‐scores, as prespecified and as expanded on elsewhere (Rubio‐Codina et al., 2016). For the Bayley‐III, we generated residuals from a linear regression of raw scores on tester dummies and standardised these relative to the age‐specific mean and standard deviation (*SD*) of the control group, both estimated nonparametrically. Because the ASQ‐3 has separate age‐specific questionnaires, we standardised scores within each age bracket. In robustness analysis, we estimated impacts on raw and composite (externally standardised) scores.

We preregistered three secondary outcomes: the quality of the home environment, maternal knowledge of child development and maternal time spent on stimulation activities with children. We dropped the latter outcome given poor data quality, as indicated by the majority of time reports summing to <20 hr. We additionally report exploratory impacts on maternal depressive symptoms but note that since this analysis is exploratory, results should not be interpreted causally but rather to inform questions for future work.

Secondary outcomes were measured in the household survey at baseline and endline. The quality of the home environment was assessed using two scales of UNICEF's Family Care Indicators (FCI): the variety of play materials the child uses, collected by observation; and the variety of play activities the child engaged in with an adult over the preceding three days, collected by maternal report (Kariger et al., 2012). Items were combined into an index using a two‐parameter item response theory model. Maternal knowledge of child development was assessed using 17 items adapted from previous scales measuring knowledge of child development (Chang et al., 2015; MacPhee, 1981) which are listed in the baseline report (Andrew et al., 2015). Maternal depressive symptoms were assessed using short versions of the Center for Epidemiologic Studies depression scale (CES‐D), with 6 items at baseline and 10 items (Andresen et al., 1994) at endline. Since item responses used Likert scales, indexes for both were constructed using exploratory factor analysis. All tools were translated into Oriya and extensively piloted.

No financial payments were made to participants. However, as a token of appreciation for participating in the Bayley‐III assessment, children received a small book and caregivers received a metal plate.

### Statistical analysis

Our analysis followed a pre‐analysis plan which is available from the trial registries (ISRCTN89476603 and AEARCTR‐0000169). Assuming 10% attrition and an intracluster correlation of 0.04, as in similar studies (Attanasio et al., 2014), we estimated minimum detectable effects with power of 80%, using a difference in means test, of 0.29 *SD*. This magnitude is similar to the mean effect size on cognition found in a recent literature review of early childhood interventions (Nores & Barnett, 2010).

For primary and secondary outcomes, we used intention‐to‐treat analysis by calculating the difference in means between the treatment and control groups. We present both unadjusted estimates of the intervention's effect on child development and estimates adjusted for a set of prespecified baseline controls. Controls were chosen as characteristics we judged would predict Bayley‐III scores: baseline development, as measured by the problem solving, communication and fine motor subscales of the ASQ‐3; firstborn; child gender; and mothers' education. All estimates for child development outcomes implicitly control for age and tester effects through the standardisation procedure. Since all outcome measures were scaled to have zero mean and unit variance in the control group, estimates can be interpreted in *SD* units relative to the control group. In prespecified heterogeneity analysis, we examined effects by gender by regressing outcome measures on indicators for treatment, for gender and the two interacted.

To inform future research on targeting and design of early years' interventions, we additionally report heterogeneity by maternal education, baseline development as measured by the ASQ‐3 and by baseline stunting. We note that these analyses were not prespecified and so results should not be interpreted causally.

95% confidence intervals (CIs) and two‐sided *p*‐values were calculated using a coefficient bootstrap (5,000 replications), resampling clusters within each randomisation strata. We also show analogous *p*‐values corrected for multiple hypotheses testing across all outcome measures being tested simultaneously using the Romano–Wolf stepdown procedure (Romano & Wolf, 2005; *stepdown p*). These are interpreted as the probability of incorrectly rejecting at least one of the hypotheses being tested and thus finding a false positive (Familywise Error Rate). All primary outcomes were tested simultaneously as were, separately, secondary outcomes. When estimating heterogeneous effects, we corrected *p*‐values across all outcomes and both groups. As an alternative method of dealing with multiple hypothesis testing (Anderson, 2008), we present impacts on a summary ‘Bayley‐III factor index’, constructed as the first factor from an exploratory factor analysis of the four Bayley‐III Z‐scores.

## Results

**Figure 1:**
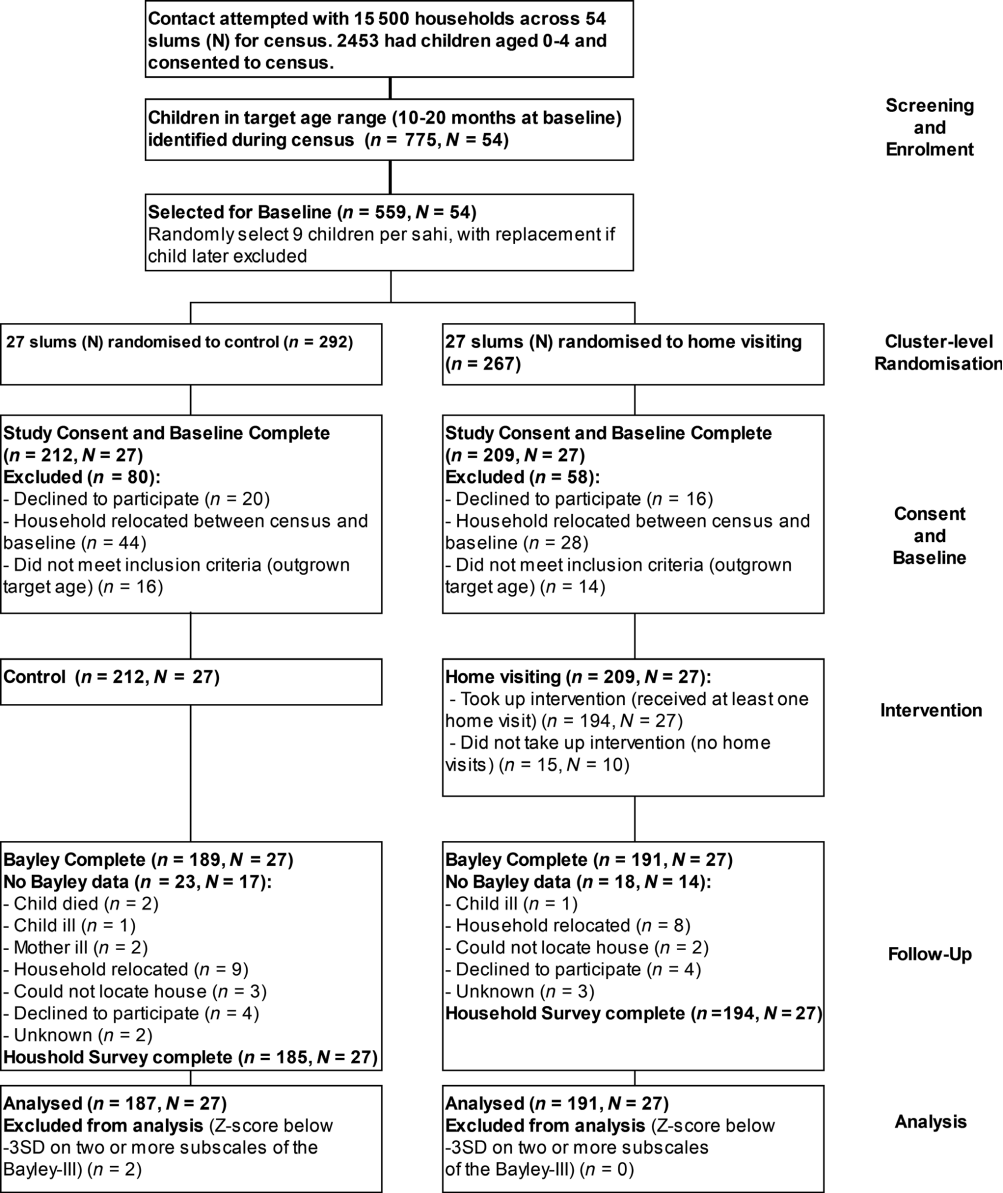
Study flow diagram

Figure 1 shows the trial flow diagram. Table 1 provides descriptive statistics of the baseline sample and demonstrates the economic disadvantage of sample households, 46% of whom lived below the poverty line. Attrition between baseline and endline, at 11.8% in control and 8.6% in treatment, was not significantly different between groups (*p* = .234). Most attrition was due to temporary or permanent relocation and refusal, at 1.89% (1.91%) in the control (treatment) group, was uncommon (Figure 1). In both treatment arms, lost subjects did not significantly differ from those included in their baseline characteristics (Table S2). Amongst those included in the analysis baseline characteristics, except maternal education, were well‐balanced across treatment and control (Table S3) with the difference in maternal education not being significant once *p*‐values were corrected for multiple testing (*stepdown p* = .180). Intracluster correlations of primary outcomes within slums varied between 0.049 (expressive language) and 0.120 (cognition).

**Table 1:** Baseline characteristics by randomisation status

	Control (*n* = 212)	Treatment (*n* = 209)
Age in months	15.112 (3.234)	14.721 (3.066)
Male, %	0.476 (0.501)	0.565 (0.497)
Firstborn, %	0.467 (0.500)	0.478 (0.501)
Mother's years of education	6.722 (3.842)	8.091 (3.359)
Household asset index *Z*‐score	−0.125 (0.929)	0.133 (0.925)
Length‐for‐age WHO *Z*‐score	−1.142 (1.257)	−0.931 (1.297)
Weight‐for‐length WHO *Z*‐score	−0.574 (1.204)	−0.472 (1.140)
ASQ‐3 problem solving *Z*‐score	−0.000 (1.000)	0.057 (0.982)
ASQ‐3 communication *Z*‐score	−0.000 (1.000)	−0.032 (1.099)
ASQ‐3 fine motor *Z*‐score	−0.000 (1.000)	−0.073 (1.074)
Maternal knowledge of child development *Z*‐score	0.000 (1.000)	−0.063 (0.915)
Quality of home environment *Z*‐score	0.000 (1.000)	0.112 (1.157)
Maternal depressive symptoms *Z*‐score	−0.000 (1.000)	−0.061 (1.072)
Below Urban Poverty Line^a^	0.486 (0.501)	0.483 (0.501)
Income (Rs) per Capita per Day^b^	98.446 (218.178)	109.747 (218.092)
Roof made from metal sheet/thatch/polyethylene	0.462 (0.500)	0.388 (0.488)
House has dirt floor	0.052 (0.222)	0.057 (0.233)
House has piped water connection	0.575 (0.495)	0.598 (0.491)
Household has electricity connection	0.986 (0.118)	0.986 (0.119)
Household owns a fridge	0.308 (0.463)	0.402 (0.491)

Data are mean (*SD*) or % (*n*). *Z*‐scores scaled to have zero mean and unit variance in the control group.

a Urban poverty line as defined by the Rangarajan committee is Rs. 47 per household member per day.

b The average exchange rate during the baseline survey (November/December 2013) was Rs. 62/USD.

Intervention take‐up and compliance was high: 92.8% of children allocated to treatment (and 94.76% for those for whom we have Bayley‐III data) received at least one visit and, of those, the average number received was 54.3, amounting to three per month or 87% of those scheduled (Table S4). Of the visits that were planned but did not occur, the most common reasons were that the child or mother was not available (68%), usually due to visiting relatives or sickness, or that the HV was not available (22%), typically for similar reasons (Table S5). Take‐up and number of visits were not significantly related to baseline characteristics.

Table 2 shows the intervention improved cognition by 0.349 *SD* (*p* = .005, *stepdown p* = .017). Effects on receptive language (0.224 *SD*; *p* = .099 *stepdown p* = .184) and expressive language (0.192 *SD*; *p* = .085, *stepdown p* = .184) were marginally significant. While there was no significant intervention impact on fine motor development, the overall Bayley‐III factor index (eigenvalue = 1.8, factor loadings = [0.64–0.70]) improved by 0.301 *SD* (*p* = .032). These estimates were similar to estimates controlling for prespecified baseline characteristics: effects on the overall Bayley‐III factor index remained significant at the 10% level (*p* = .098) and again were largely driven by the effect on cognition (*p* = .016, *stepdown p* = .053). Results were robust to using raw and composite (externally standardised) scores (Tables S6 and S7) and to controlling for additional baseline characteristics and the randomisation stratifier (Tables S8–S10). Mean composite scores from the control group indicate that study children lagged substantially behind the American norms (mean of 100) for cognition but appeared on par for language and motor development (Table S7).

**Table 2:** Estimated effects of home visiting on child development

	Unadjusted for baseline controls				Adjusted for prespecified baseline controls				*N*
	Effect size	95% CI	*p*‐Value	Stepdown *p*‐value	Effect size	95% CI	*p*‐value	Stepdown *p*‐value	
Bayley‐III Z‐Scores									
Cognition	0.349	(0.100, 0.592)	.005	.017	0.293	(0.054, 0.518)	.016	.053	377
Receptive language	0.224	(−0.047, 0.489)	.099	.184	0.180	(−0.064, 0.417)	.146	.319	378
Expressive language	0.192	(−0.024, 0.415)	.085	.184	0.111	(−0.090, 0.303)	.297	.456	369
Fine motor	0.111	(−0.133, 0.358)	.358	.358	0.067	(−0.167, 0.296)	.574	.574	378
Bayley‐III Factor Index	0.301	(0.027, 0.576)	.032		0.214	(−0.040, 0.455)	.098		368

Estimates expressed in *SD*s of the control group.

Table 3 shows that boys significantly improved in cognition and receptive language while the intervention did not lead to a significant improvement for girls in any domain. The overall Bayley factor improved by 0.446 *SD* (*p* = .028, *stepdown p* = .053) for boys while the corresponding effect for girls was 0.152 *SD* (*p* = .334, *stepdown p* = .334). In the control group, girls outperformed boys across all domains, a pattern that treatment reversed.

**Table 3:** Estimated effects of home visiting on child development by gender

	Control mean	Effect size	95% CI	*p*‐Value	Stepdown *p*‐value	Control mean	Effect size	95% CI	*p*‐Value	Stepdown *p*‐value	Test of difference between groups		*N*
											*p*‐Value	Step down *p*‐value	
Gender	Girls (*N* = 180)					Boys (*N* = 198)							
Cognition	0.057	0.253	(−0.095, 0.614)	.155	.567	−0.060	0.440	(0.138, 0.737)	.003	.022	.371	.712	377
Receptive language	0.176	−0.054	(−0.338, 0.225)	.715	.781	−0.186	0.490	(0.110, 0.853)	.011	.057	.014	.042	378
Expressive language	0.020	0.148	(−0.073, 0.372)	.185	.594	−0.022	0.233	(−0.151, 0.616)	.229	.603	.697	.904	369
Fine motor	0.081	0.151	(−0.164, 0.495)	.390	.744	−0.086	0.102	(−0.234, 0.424)	.531	.781	.828	.904	378
Bayley‐III Factor Index	0.097	0.152	(−0.149, 0.472)	.334	.334	−0.105	0.446	(0.051, 0.833)	.028	.053	.195		368

Estimates expressed in *SD*s of the control group.

Exploratory heterogeneity analysis, which we do not interpret causally as it was not prespecified, suggests the intervention may have had larger benefits for children of mothers with at least 8 years of education, corresponding to upper primary school completion, and for children who were stunted at baseline, while no differences in impacts were observed by baseline child development (Table S11).

The intervention improved the quality of the home environment by 0.318 *SD* (*p* = .007, *stepdown p* = .016; Table 4), driven by an increase in both play activities and play materials (Table S12), but there was no improvement in maternal knowledge of child development. Maternal depressive symptoms, which we include as an exploratory outcome, declined by 0.266 *SD* (*p* = .013; Table S13). All secondary outcomes correlated with child development in the expected directions (Table S14).

**Table 4:** Estimated effects of home visiting on secondary outcomes

	Effect size	95% CI	*p*‐Value	Stepdown *p*‐value	*N*
Maternal knowledge of child development	0.070	(−0.217, 0.344)	.626	.626	350
Quality of the home environment	0.318	(0.092, 0.551)	.007	.016	361

Estimates expressed in *SD*s of the control group.

## Discussion

This effectiveness trial showed that a home‐visiting stimulation intervention improved the development of disadvantaged young children in Indian urban slums. The magnitude of these effects was roughly equal to the average difference between children of mothers with and without complete primary school.

The study was the first step towards creating a scalable and effective intervention for India. The positive findings demonstrate for the first time that, with appropriate cultural adaptations, the Jamaican Reach Up model can be successfully delivered in an Indian city slum. Moreover, they indicate that the model is effective when run and managed by the Cuttack branch of a national NGO, using local women hired from an abundant labour pool as frontline workers and as trainers and supervisors. This suggests that the model could feasibly be implemented at a much larger scale within the existing structures of Pratham, another large NGO, or the government.

The model complements existing early childhood government policy in India, where the emphasis on stimulation begins at age three, in two ways. First, the age targeted means children would graduate from home visits into existing Anganwadi preschools. We have subsequently held discussions with ICDS about how to improve the integration of graduates with ICDS and are presently piloting an intervention in Anganwadi centres to ensure children graduate into high‐quality services. Second, as Anganwadi workers already visit homes of younger children to monitor their physical health, the possibility of using Anganwadi centres as a base for the Reach Up intervention could be explored. It would be critical, however, to ensure that such an intervention received additional resources so that it would not detract from the existing work of Anganwadi workers.

Given this was the first evaluation of this model in India and given concerns that a lack of support for home visitors may have reduced the quality of visits in Colombia (Andrew et al., 2018), we invested heavily in home visitors' support and training. Mentors initially trained the home visitors for four weeks and then met with visitors weekly throughout the intervention and provided refresher trainings. While this level of support increases costs, it may be necessary to ensure sufficient quality in delivery given the model is designed for visitors with no specific background in early childhood. The larger effect sizes compared to Colombia suggest that the increased attention paid to supporting home visitors may have helped to sustain quality in a scalable model (Andrew et al., 2018; Attanasio et al., 2014). Nevertheless, maintaining quality at scale and within resource constraints remains a critical question. Careful implementation research assessing the impact of compromises to intervention design in aid of scale and affordability, such as reducing the intensity of support, on child development is required to gauge whether some such compromises undermine their ultimate objective of taking effective interventions to scale.

While larger than Colombia (0.26 *SD*; Attanasio et al., 2014) and similar to Bangladesh (0.33 *SD*; Hamadani, Huda, Khatun, & Grantham‐McGregor, 2006), at 0.349 *SD*s, the impacts on children's cognitive development were smaller than in the Jamaican studies (0.88 *SD*–1.7 *SD*; Grantham‐McGregor, Powell, Walker, & Himes, 1991; Grantham‐McGregor & Smith, 2016). The Jamaican studies worked with fewer children; and in Jamaica and Bangladesh, quality was more tightly monitored by researchers. These studies also targeted malnourished children whereas in this study all slum‐dwelling families were potentially eligible. Exploratory heterogeneity analysis suggested this targeting might be important in explaining differences in effect sizes; this is a key question for future research.

The effects were substantially larger for boys than girls. This reversed the pattern observed in the control group where girls outperformed boys. Son preference is well documented in Northern India, and in particular in urban areas, where female foetuses are more likely to be aborted, girls are breastfed less and receive less parental child care (Barcellos et al., 2014). If this affected caregivers or HVs response to the intervention, it could have caused the differential effect. However, girls received no fewer visits and the effect on their home environments was similar. Studies in the USA show that boys and girls may respond differently depending on the outcome (Duncan & Magnuson, 2013). Intervention material deliberately featured girls more than boys but more needs to be done to ensure girls benefit.

Exploratory analysis suggested potentially important hypotheses for further work. First, our results suggested that benefits were substantially larger for children whose mothers had more education. If this finding is confirmed by future studies, then modifying the curriculum to make it more accessible to less educated mothers should be considered. Second, our findings suggested benefits were substantially higher for children who were stunted at baseline; a confirmation of this would suggest that targeting interventions at malnourished children might be appropriate. Third, exploratory analysis indicated a reduction in maternal depressive symptoms, similar to findings in Baker‐Henningham et al. (2005). Although not specifically targeted, social support to mothers provided by HVs might be responsible.

Strengths of the study are that the intervention was run and managed through a local branch of a national NGO, drew on an abundant labour pool and is complementary to existing childhood services in India. Further strengths are the randomised study design and the quality of the primary outcome measures. Despite demonstrating that interventions can be run through local institutions, the current costs of this intervention remain a substantial investment for governments and NGOs in LMICs and ongoing work is assessing the potential of group sessions, reduced intensity and better targeting to further increase scalability. Other limitations are the limited geographic area and the use of a screener test for the assessment of baseline child development.

## Conclusions

A home‐visiting parenting programme was effective at improving disadvantaged young children's development. In considering if and how to scale such a programme, either through a large NGO or through India's national early childhood programme, the ICDS, maintaining fidelity will be key. How changes to the intervention model deemed necessary for scaling impact its effectiveness should be explicitly evaluated.

## Supporting information


**Table S1.** Intervention costs.
**Table S2.** Baseline characteristics by attrition status.
**Table S3.** Baseline characteristics by randomization status for non‐attritors.
**Table S4.** Number of home visits received by children allocated to treatment group.
**Table S5.** Reasons why planned visits did not occur.
**Table S6.** Treatment effects on Bayley‐III raw scores.
**Table S7.** Treatment effects on Bayley‐III composite (externally standardized) scores.
**Table S8.** Estimated effects of home visiting on child development (alternative controls).
**Table S9.** Estimated effects of home visiting on child development (alternative controls).
**Table S10.** Estimated effects of home visiting on child development (controlling for stratifier).
**Table S11.** Exploratory heterogeneity analysis.
**Table S12.** Estimated effects of home visiting on quality of the home environment (by subscale).
**Table S13.** Estimated effects of maternal depressive symptoms.
**Table S14.** Correlations between child development and secondary outcomes.
