# Supplementary material for: Effects of a scalable home‐visiting intervention on child development in slums of urban India: evidence from a randomised controlled trial
Source: J Child Psychol Psychiatry. 2019 Dec 3;61(6):644–52. doi: 10.1111/jcpp.13171 (PMC7242140; doi:10.1111/jcpp.13171)
Supplement: Supplementary file 1 — Table S1. Intervention costs. Table S2. Baseline characteristics by attrition status. Table S3. Baseline characteristics by randomization status for non‐attritors. Table S4. Number of home visits received by children allocated to treatment group. Table S5. Reasons why planned visits did not occur. Table S6. Treatment effects on Bayley‐III raw scores. Table S7. Treatment effects on Bayley‐III composite (externally standardized) scores. Table S8. Estimated effects of home visiting on child development (alternative controls). Table S9. Estimated effects of home visiting on child development (alternative controls). Table S10. Estimated effects of home visiting on child development (controlling for stratifier). Table S11. Exploratory heterogeneity analysis. Table S12. Estimated effects of home visiting on quality of the home environment (by subscale). Table S13. Estimated effects of maternal depressive symptoms. Table S14. Correlations between child development and secondary outcomes. [file JCPP-61-644-s001.docx]

**Supporting information –** **Effects of a scalable home visiting intervention on child development in slums of urban India: evidence from a randomised controlled trial – by Andrew *et al*.**

**Table S1.** Intervention costs.

| **INTERVENTION (GBP)** |  | **GBP - 18 months** | **US$ - 18 months** | **US$ - 12 months** |
| --- | --- | --- | --- | --- |
| Salaries | Mentors | 9,846 | 15,579 | 10,386 |
|  | Facilitators | 13,185 | 20,862 | 13,908 |
| Training of mentors and facilitators |  | 5,807 | 9,188 | 6,126 |
| Intervention materials |  | 3,978 | 6,294 | 4,196 |
| Community meetings |  | 390 | 617 | 411 |
| Total |  |  | 52,541 | 35,027 |
| **Yearly cost per treatment child** |  |  | 251 | 168 |

US$ conversions using 2014 average exchange rate of 0.632 GBP/US$. Treatment children refer to all children randomised into the treatment group.

**Table S2.** Baseline characteristics by attrition status.

|  | | **Treatment (n=209)** | | | | | | **Control (n=212)** | | | | | |
| --- | --- | --- | --- | --- | --- | --- | --- | --- | --- | --- | --- | --- | --- |
|  | | **Non-Attriters**  **(n=191)** | | **Attriters**  **(n=18)** | | **P-value** | **Stepdown**  **P-value** | **Non-Attriters**  **(n=187)** | | **Attriters**  **(n=25)** | | **P-value** | **Stepdown**  **P-value** |
| Age in months | | 14.733 | 3.062 | 14.593 | 3.200 | 0.849 | 1.000 | 15.132 | 3.199 | 14.960 | 3.553 | 0.805 | 1.000 |
| Male, | | 0.560 | 0.498 | 0.611 | 0.502 | 0.649 | 1.000 | 0.487 | 0.501 | 0.400 | 0.500 | 0.377 | 0.992 |
| Firstborn, | | 0.476 | 0.501 | 0.500 | 0.514 | 0.849 | 1.000 | 0.465 | 0.500 | 0.480 | 0.510 | 0.896 | 1.000 |
| Mother's years of education | | 8.016 | 3.379 | 8.889 | 3.123 | 0.154 | 0.775 | 6.797 | 3.730 | 6.160 | 4.643 | 0.533 | 0.997 |
| Household asset index Z-score | | 0.127 | 0.935 | 0.202 | 0.826 | 0.645 | 1.000 | -0.086 | 0.921 | -0.417 | 0.961 | 0.114 | 0.723 |
| Length-for-age WHO Z-score | | -0.874 | 1.289 | -1.517 | 1.261 | 0.020 | 0.238 | -1.142 | 1.254 | -1.147 | 1.302 | 0.981 | 1.000 |
| Weight-for-length WHO Z-score | | -0.460 | 1.097 | -0.599 | 1.562 | 0.724 | 1.000 | -0.577 | 1.188 | -0.546 | 1.346 | 0.887 | 1.000 |
| ASQ-3 problem solving Z-score | | 0.060 | 0.973 | 0.031 | 1.100 | 0.923 | 1.000 | 0.040 | 0.988 | -0.296 | 1.063 | 0.084 | 0.627 |
| ASQ-3 communication Z-score | | -0.033 | 1.083 | -0.018 | 1.290 | 0.959 | 1.000 | 0.069 | 0.951 | -0.513 | 1.212 | 0.020 | 0.247 |
| ASQ-3 fine motor Z-score | | -0.027 | 1.058 | -0.563 | 1.146 | 0.064 | 0.498 | 0.036 | 0.987 | -0.273 | 1.074 | 0.099 | 0.680 |
| Maternal knowledge of child development Z-score | | 0.084 | 1.149 | 0.411 | 1.234 | 0.307 | 0.949 | 0.018 | 0.989 | -0.136 | 1.090 | 0.468 | 0.997 |
| Quality of home environment Z-score | | -0.055 | 0.920 | -0.151 | 0.880 | 0.503 | 0.997 | 0.004 | 0.997 | -0.031 | 1.045 | 0.875 | 1.000 |
| Maternal depressive symptoms Z-score | | -0.085 | 1.047 | 0.200 | 1.320 | 0.342 | 0.974 | 0.005 | 1.000 | -0.038 | 1.021 | 0.834 | 1.000 |
| Below Urban Poverty Line^a^ | | 0.482 | 0.501 | 0.500 | 0.514 | 0.869 | 1.000 | 0.460 | 0.500 | 0.680 | 0.476 | 0.004 | 0.069 |
| Income (Rs) per Capita per Day^b^ | | 109.90 | 220.19 | 108.06 | 199.21 | 0.965 | 1.000 | 101.85 | 223.23 | 73.018 | 177.57 | 0.468 | 0.997 |
| Roof made from metal sheet/thatch/polyethylene | | 0.403 | 0.492 | 0.222 | 0.428 | 0.078 | 0.516 | 0.476 | 0.501 | 0.360 | 0.490 | 0.144 | 0.815 |
| House has dirt floor | | 0.058 | 0.234 | 0.056 | 0.236 | 0.956 | 1.000 | 0.053 | 0.226 | 0.040 | 0.200 | 0.718 | 1.000 |
| House has piped water connection | | 0.581 | 0.495 | 0.778 | 0.428 | 0.061 | 0.498 | 0.572 | 0.496 | 0.600 | 0.500 | 0.817 | 1.000 |
| Household has electricity connection | | 0.984 | 0.125 | 1.000 | 0.000 | 0.076 | 0.498 | 0.984 | 0.126 | 1.000 | 0.000 | 0.043 | 0.502 |
| Household owns a fridge | | 0.393 | 0.490 | 0.500 | 0.514 | 0.373 | 0.975 | 0.312 | 0.464 | 0.280 | 0.458 | 0.762 | 1.000 |
|  | Data are mean (SD). Attriters refers to children who had complete baseline data but for whom we don’t have endline data. Z-scores scaled to have zero mean and unit variance in the control group. ^a^Urban poverty line as defined by the Rangarajan committee is Rs. 47 per household member per day. ^b^The average exchange rate during the baseline survey (November/December 2013) was Rs. 62/USD. | | | | | | | | | | | | |

**Table S3.** Baseline characteristics by randomization status for non-attritors.

|  | **Control**  **(n = 187)** | | | **Treatment**  **(n = 191)** | | | **P-value** | | **Stepdown**  **P-value** | |
| --- | --- | --- | --- | --- | --- | --- | --- | --- | --- | --- |
| Age in months | 15.132 | (3.199) | 14.733 | | (3.062) | 0.215 | | 0.951 | |  |
| Male, % | 48.7 | (91) | 56.0 | | (107) | 0.227 | | 0.951 | |  |
| Firstborn, % | 46.5 | (87) | 47.6 | | (100) | 0.814 | | 1.000 | |  |
| Mother's years of education | 6.797 | (3.730) | 8.016 | | (3.379) | 0.018 | | 0.246 | |  |
| Household asset index Z-score | -0.000 | (1.000) | 0.231 | | (1.013) | 0.109 | | 0.767 | |  |
| Length-for-age WHO Z-score | -1.142 | (1.254) | -0.874 | | (1.289) | 0.059 | | 0.560 | |  |
| Weight-for-length WHO Z-score | -0.577 | (1.188) | -0.460 | | (1.097) | 0.307 | | 0.980 | |  |
| ASQ-3 problem solving Z-score | 0.000 | (1.000) | 0.020 | | (0.986) | 0.848 | | 1.000 | |  |
| ASQ-3 communication Z-score | 0.000 | (1.000) | -0.107 | | (1.139) | 0.324 | | 0.980 | |  |
| ASQ-3 fine motor Z-score | 0.000 | (1.000) | -0.064 | | (1.072) | 0.525 | | 0.998 | |  |
| ASQ-3 factor Z-score | 0.000 | (1.000) | -0.059 | | (1.087) | 0.586 | | 0.999 | |  |
| Maternal knowledge of child development Z-score | 0.000 | (1.000) | -0.059 | | (0.923) | 0.509 | | 0.998 | |  |
| Quality of home environment Z-score | 0.000 | (1.000) | 0.066 | | (1.162) | 0.642 | | 0.999 | |  |
| Maternal depressive symptoms Z-score | 0.000 | (1.000) | -0.090 | | (1.047) | 0.373 | | 0.987 | |  |
| Below Urban Poverty Line^a^ | 0.460 | 0.500 | 0.482 | | 0.501 | 0.715 | | 1.000 | |  |
| Income (Rs) per Capita per Day^b^ | 101.845 | 223.232 | 109.899 | | 220.187 | 0.741 | | 1.000 | |  |
| Roof made from metal sheet/thatch/polyethylene | 0.476 | 0.501 | 0.403 | | 0.492 | 0.374 | | 0.987 | |  |
| House has dirt floor | 0.053 | 0.226 | 0.058 | | 0.234 | 0.906 | | 1.000 | |  |
| House has piped water connection | 0.572 | 0.496 | 0.581 | | 0.495 | 0.922 | | 1.000 | |  |
| Household has electricity connection | 0.984 | 0.126 | 0.984 | | 0.125 | 0.917 | | 1.000 | |  |
| Household owns a fridge | 0.312 | 0.464 | 0.393 | | 0.490 | 0.205 | | 0.949 | |  |

Data are mean (SD) or % (n). Z-scores scaled to have zero mean and unit variance in the control group. ^a^Urban poverty line as defined by the Rangarajan committee is Rs. 47 per household member per day. ^b^The average exchange rate during the baseline survey (November/December 2013) was Rs. 62/USD.

**Table S4.** Number of home visits received by children allocated to treatment group.

|  | **All Treatment Group (n=209)** | | **Analysis Sample (i.e. non-missing Bayley-III data) (n=191)** | |
| --- | --- | --- | --- | --- |
|  | **Frequency** | **Percent** | **Frequency** | **Percent** |
| **No visits** | 15 | 7.18 | 10 | 5.24 |
| **1-20 visits** | 18 | 8.61 | 11 | 5.76 |
| **21-40 visits** | 13 | 6.22 | 12 | 6.28 |
| **41-60 visits** | 75 | 35.89 | 72 | 37.7 |
| **61+ visits** | 88 | 42.11 | 86 | 45.03 |
| **Total** | **209** | **100** | **191** | **100** |
| **Mean (SD) number of visits** | 50.36 (22.29) | | 53.27 (19.84) | |
| **Mean (SD) visits conditional at least one visit** | 54.26 (17.97) | | 56.21 (15.78) | |

This table presents the distribution of home visits received by children in the treatment group (columns 1-2) as documented in administrative records. Columns 3-4 show this distribution for the children whom we have Bayley-III data for. The intervention started on a rolling basis across sahis so the scheduled number of visits varied between 54 and 66. This accounts for 8-10 visits lost over the intervention period due to festivals, trainings and toy-making workshops.

**Table S5.** Reasons why planned visits did not occur.

| Child or mother unavailable due to sickness or personal reasons | 68% |
| --- | --- |
| Home visitor unavailable due to sickness or personal reasons | 22% |
| Festival | 6% |
| Home visitor at training workshop | 2% |
| Other | 2% |

**Table S6.** Treatment effects on Bayley-III raw scores.

|  | **Effect Size** | **95% CI** | **P-value** | **Stepdown**  **P-value** | **N** | **Control**  **Mean** | **(SD)** | **Treatment Mean** | **(SD)** | |
| --- | --- | --- | --- | --- | --- | --- | --- | --- | --- | --- |
| Cognition | 1.300 | (0.302, 2.329) | 0.013 | 0.037 | 377 | 67.02 | (4.56) | 68.19 | (4.41) | |
| Receptive language | 0.829 | (-0.182, 1.849) | 0.105 | 0.226 | 378 | 33.80 | (3.86) | 34.58 | (4.28) | |
| Expressive language | 0.838 | (-0.195, 2.070) | 0.144 | 0.234 | 369 | 37.24 | (5.08) | 38.52 | (6.45) | |
| Fine motor | 0.333 | (-0.628, 0.380) | 0.504 | 0.504 | 378 | 43.88 | (4.45) | 44.11 | (4.66) | |
| Estimates of the impact of the home visiting intervention on Bayley-III raw scores (i.e. without standardizing for age and testers). Estimates control interviewer effects and age in months to ensure equivalence to main results on age- and tester-standardised scores. | | | | | | | | | |  |

**Table S7.** Treatment effects on Bayley-III composite (externally standardized) scores.

|  | **Effect Size** | **95% CI** | **P-value** | **Stepdown**  **P-value** | **N** | **Control**  **Mean (SD)** | | **Treatment**  **Mean (SD)** | |
| --- | --- | --- | --- | --- | --- | --- | --- | --- | --- |
| Cognition | 2.185 | (0.601, 3.928) | 0.010 | 0.027 | 377 | 89.01 | (7.09) | 91.34 | (7.14) |
| Language | 2.108 | (-0.322, 4.860) | 0.110 | 0.179 | 369 | 101.66 | (10.52) | 104.86 | (11.97) |
| Motor | -0.697 | (-4.014, 2.901) | 0.684 | 0.684 | 378 | 97.38 | (14.54) | 97.50 | (13.11) |
| Estimates of the impact of the home visiting intervention on Bayley-III composite scores constructed in accordance with the test’s manual. Note that receptive and expressive language, and fine and gross motor are combined in creating composite scores. Estimates control for interviewer effects squared to ensure equivalence to main results on age- and tester-standardised scores. | | | | | | | | | |

**Table S8.** Estimated effects of home visiting on child development (alternative controls).

|  |  | **Adjusted for Suggested Controls** | | | | |  |  |
| --- | --- | --- | --- | --- | --- | --- | --- | --- |
|  |  | **Effect Size** | **95% CI** | **P-value** | **Stepdown**  **P-value** | **N** | | |
| **Bayley-III Z-Scores** |  |  |  |  |  |  | | |
| Cognition |  | 0.294 | (0.061 to 0.520) | 0.014 | 0.047 | 377 | | |
| Receptive language |  | 0.166 | (-0.068 to 0.386) | 0.160 | 0.341 | 378 | | |
| Expressive language |  | 0.095 | (-0.097 to 0.286) | 0.369 | 0.540 | 369 | | |
| Fine motor |  | 0.079 | (-0.150 to 0.303) | 0.495 | 0.540 | 378 | | |
| **Bayley-III Factor Index** |  | 0.211 | (-0.040 to 0.449) | 0.097 |  | 368 | | |

Estimates expressed in SDs of the control group. Estimates control for child gender, firstborn, mother’s education, baseline development, asset index, stunting.

**Table S9.** Estimated effects of home visiting on child development (controlling for age).

|  |  | **Adjusted for Suggested Controls** | | | | |  |  |
| --- | --- | --- | --- | --- | --- | --- | --- | --- |
|  |  | **Effect Size** | **95% CI** | **P-value** | **Stepdown**  **P-value** | **N** | | |
| **Bayley-III Z-Scores** |  |  |  |  |  |  | | |
| Cognition |  | 0.357 | (0.106 to 0.598) | 0.004 | 0.014 | 377 | | |
| Receptive language |  | 0.223 | (-0.052 to 0.486) | 0.102 | 0.172 | 378 | | |
| Expressive language |  | 0.196 | (-0.021 to 0.415) | 0.081 | 0.172 | 369 | | |
| Fine motor |  | 0.112 | (-0.133 to 0.356) | 0.360 | 0.360 | 378 | | |
| **Bayley-III Factor Index** |  | 0.304 | (0.024 to 0.573) | 0.030 |  | 368 | | |

Estimates expressed in SDs of the control group. Estimates control for child’s age.

**Table S10.** Estimated effects of home visiting on child development (controlling for stratifier).

|  |  | **Adjusted for Suggested Controls** | | | | |  |  |
| --- | --- | --- | --- | --- | --- | --- | --- | --- |
|  |  | **Effect Size** | **95% CI** | **P-value** | **Stepdown**  **P-value** | **N** | | |
| **Bayley-III Z-Scores** |  |  |  |  |  |  | | |
| Cognition |  | 0.348 | (0.099 to 0.593) | 0.005 | 0.019 | 377 | | |
| Receptive language |  | 0.225 | (-0.044 to 0.493) | 0.098 | 0.189 | 378 | | |
| Expressive language |  | 0.194 | (-0.024 to 0.423) | 0.087 | 0.189 | 369 | | |
| Fine motor |  | 0.113 | (-0.131 to 0.358) | 0.359 | 0.359 | 378 | | |
| **Bayley-III Factor Index** |  | 0.302 | (0.029 to 0.581) | 0.033 |  | 368 | | |

Estimates expressed in SDs of the control group. Estimates control for randomisation stratifier.

**Table S11.** Exploratory heterogeneity analysis.

|  | **Control**  **Mean** | **Effect**  **Size** | **SE** | **P-value** | **Stepdown P-value** | **Control**  **Mean** | **Effect Size** | **SE** | **P-value** | **Stepdown P-value** | **Test of Difference between Groups** | | **N** |  |
| --- | --- | --- | --- | --- | --- | --- | --- | --- | --- | --- | --- | --- | --- | --- |
|  |  |  |  |  |  |  |  |  |  |  | **P- value** | **Step down P-value** |  |  |
| Panel A. **Baseline Development** | **Baseline ASQ-3 factor index below median**  **(N =185)** | | | | | **Baseline ASQ-3 factor index above median**  **(N =193)** | | | | |  |  |  | |
| Cognition | -0.240 | 0.398 | 0.146 | 0.003 | 0.021 | 0.223 | 0.314 | 0.168 | 0.031 | 0.134 | 0.662 | 0.956 | 377 |  |
| Receptive language | -0.169 | 0.148 | 0.160 | 0.182 | 0.375 | 0.157 | 0.310 | 0.159 | 0.024 | 0.123 | 0.353 | 0.816 | 378 |  |
| Expressive language | -0.191 | 0.191 | 0.143 | 0.095 | 0.249 | 0.173 | 0.199 | 0.125 | 0.054 | 0.194 | 0.956 | 0.993 | 369 |  |
| Fine motor | -0.171 | 0.108 | 0.126 | 0.196 | 0.375 | 0.158 | 0.126 | 0.166 | 0.227 | 0.375 | 0.913 | 0.993 | 378 |  |
| **Bayley-III Factor Index** | -0.239 | 0.288 | 0.150 | 0.030 | 0.045 | 0.217 | 0.317 | 0.161 | 0.024 | 0.045 | 0.836 |  | 368 |  |
| **Panel B. Maternal Education** | **Less than 8^th^ Standard (N =157)** | | | | | **8^th^ Standard or more (N =221)** | | | | |  |  |  | |
| Cognition | -0.184 | 0.152 | 0.155 | 0.165 | 0.376 | 0.178 | 0.369 | 0.153 | 0.008 | 0.053 | 0.274 | 0.466 | 377 |  |
| Receptive language | -0.138 | -0.095 | 0.161 | 0.727 | 0.868 | 0.134 | 0.327 | 0.155 | 0.016 | 0.101 | 0.026 | 0.078 | 378 |  |
| Expressive language | -0.283 | 0.036 | 0.156 | 0.394 | 0.660 | 0.280 | 0.136 | 0.113 | 0.116 | 0.348 | 0.551 | 0.551 | 369 |  |
| Fine motor | -0.107 | -0.266 | 0.144 | 0.970 | 0.970 | 0.103 | 0.258 | 0.153 | 0.051 | 0.206 | 0.003 | 0.014 | 378 |  |
| **Bayley-III Factor Index** | -0.240 | -0.025 | 0.161 | 0.556 | 0.556 | 0.238 | 0.353 | 0.148 | 0.008 | 0.019 | 0.024 |  | 368 |  |
| **Panel C. Stunting** | **Not Stunted (N=291)** | | | | | **Stunted (N=82)** | | | | |  |  |  | |
| Cognition | 0.113 | 0.204 | 0.138 | 0.072 | 0.190 | -0.366 | 0.892 | 0.229 | 0.000 | 0.000 | 0.007 | 0.021 | 377 |  |
| Receptive language | 0.129 | 0.084 | 0.138 | 0.276 | 0.385 | -0.448 | 0.653 | 0.253 | 0.004 | 0.027 | 0.028 | 0.072 | 378 |  |
| Expressive language | 0.118 | 0.112 | 0.109 | 0.154 | 0.294 | -0.412 | 0.422 | 0.255 | 0.051 | 0.178 | 0.261 | 0.261 | 369 |  |
| Fine motor | 0.114 | 0.012 | 0.130 | 0.463 | 0.463 | -0.374 | 0.498 | 0.224 | 0.017 | 0.064 | 0.042 | 0.072 | 378 |  |
| **Bayley-III Factor Index** | 0.148 | 0.158 | 0.140 | 0.129 | 0.129 | -0.506 | 0.794 | 0.266 | 0.003 | 0.004 | 0.019 |  | 368 |  |

Estimates expressed in SDs of the control group.

**Table S12.** Estimated effects of home visiting on quality of the home environment (by subscale).

|  | **Effect**  **Size** | **95% CI** | **P-value** | **Stepdown P-value** | **N** |
| --- | --- | --- | --- | --- | --- |
| Play activities | 0.334 | (0.100 to 0.574) | 0.007 | 0.013 | 361 |
| Play materials | 0.202 | (-0.043 to 0.464) | 0.115 | 0.115 | 361 |

Estimates expressed in SDs of the control group.

**Table S13.** Estimated effects of maternal depressive symptoms.

|  | **Effect**  **Size** | **95% CI** | **P-value** | **N** |
| --- | --- | --- | --- | --- |
| Maternal Depressive Symptoms (factor score) | -0.266 | (-0.475 to -0.057) | 0.013 | 348 |
| Maternal Depressive Symptoms (raw score) | -0.216 | (-0.409 to -0.021) | 0.031 | 348 |

Estimates expressed in SDs of the control group.

**Table S14.** Correlations between child development and secondary outcomes.

|  | Knowledge | Home Environment | Depressive Symptoms | Bayley factor |
| --- | --- | --- | --- | --- |
| Knowledge | 1 |  |  |  |
|  |  |  |  |  |
| Home Environment | 0.110^*^ | 1 |  |  |
|  |  |  |  |  |
| Depressive Symptoms | -0.234^***^ | -0.0197 | 1 |  |
|  |  |  |  |  |
| Bayley factor | 0.143^**^ | 0.411^***^ | -0.132^*^ | 1 |
|  |  |  |  |  |

^*^ *p* < 0.05, ^**^ *p* < 0.01, ^***^ *p* < 0.001.
